# Phylogenomic analysis of phenylalanine ammonia-lyase (PAL) multigene family and their differential expression analysis in wheat (*Triticum aestivum* L.) suggested their roles during different stress responses

**DOI:** 10.3389/fpls.2022.982457

**Published:** 2022-09-30

**Authors:** Chuang Zhan, Yiting Li, Han Li, Mengru Wang, Shuangjun Gong, Dongfang Ma, Yan Li

**Affiliations:** ^1^ Engineering Research Center of Ecology and Agricultural Use of Wetland, Ministry of Education/College of Agriculture, Yangtze University, Jingzhou, China; ^2^ Key Laboratory of Integrated Pest Management on Crop in Central China, Ministry of Agriculture/Hubei Province Key Laboratory for Control of Crop Diseases, Pest and Weeds/Institute of Plant Protection and Soil Science, Hubei Academy of Agricultural Sciences, Wuhan, China

**Keywords:** phenylalanine ammonia-lyase, biotic stresses, qRT-PCR, virus induced gene silencing, disease resistance

## Abstract

Phenylalanine ammonia-lyase (PAL) is a key enzyme in the phenylalanine metabolism pathway and plays an important role in plant growth and stress response. It has been widely reported in plants, but less studied in wheat. In this study, 54 *PAL* genes were identified in the wheat genome. Based on phylogenetic analysis, the 54 *TaPAL* genes were divided into four groups (I, II, III, and IV). Then, the expression levels of *TaPALs* under biotic stresses were analyzed by transcriptome data analysis. The results showed that 31 genes were up-regulated and one gene was down-regulated after inoculation with *Fusarium graminearum*, 11 genes were up-regulated and 14 genes were down-regulated after inoculation with *Puccinia striiformis*, and 32 up-regulated and three down-regulated genes after inoculation with powdery mildew. The expression patterns of the five *TaPALs* were further analyzed by qRT-PCR. After inoculation with *F. graminearum*, the expression levels of *five TaPALs* were up-regulated. However, the *TaPALs* (expect *TaPAL49*) were down-regulated when inoculated with *P. striiformis*. Finally, the functions of *TaPAL32* and *TaPAL42* in resistance of wheat to the stripe rust were further analyzed by virus induced gene silencing (VIGS) assays. The results showed that the disease severity of *TaPAL32* and *TaPAL42* silenced plants was higher than that of control plants at 14 days after inoculation. It indicated that these two genes played a positive role in wheat stripe rust resistance. This study provided new evidence support for the functional study of *PAL* genes in wheat, and provided potential application value for the breeding of wheat resistant varieties.

## Introduction

Phenylalanine ammonia-lyase (PAL, EC: 4.3.1.5) is the first and key rate-limiting enzyme in the phenylpropane metabolism pathway, which catalyzes the deamination of L-phenylalanine (L-Phe) to produce trans-cinnamic acid (MacDonald and D’Cunha, 2007) . PAL is firstly discovered in barley (*Hordeum vulgare*) seedlings (Koukal and Conn, 1966) , and now it is found in various plants and a few microorganisms (Costa et al., 2003; MacDonald and D’Cunha, 2007; Huang et al., 2010). To date, *PAL* genes have been identified in various plants such as *Arabidopsis* (Huang et al., 2010), *Populus trichocarpa* (Shi et al., 2013), banana (*Musa nana*) (Wang et al., 2016), rice (*Oryza sativa*) (Zeng et al., 2018), and walnut (*Juglans regia*) (Yan et al., 2019). The number of PAL proteins varies greatly in plants, but its molecular weight varies little, mainly ranging from 275 to 330 KDa (Rawal et al., 2013; Dong and Shang, 2013; Zhang and Liu, 2015). In plants, PAL proteins are highly conserved, and the sequence similarity of PAL among different species can even reach to 80% (Song and Wang, 2009). PAL family proteins generally contain PLN02457, Phe_AM_lyase, Lyase_aromatic and PAL-HAL domains. The active site of PAL enzyme exists in the PAL-HAL domain, most of them contain the “GTITASGDLVPLSYIAG” active center sequence, and ASG (Ala-Ser-Gly) is the most typical conserved region of PAL in plants (Song and Wang, 2009; Chen, 2019).

In plants, many secondary metabolites, such as anthocyanins, lignin, hormones and flavonoids, are synthesised by phenylpropane synthesis pathway. These secondary metabolism plays an important role in plant growth, development and environmental adaptation. (Blake et al., 2012). As the rate-limiting enzyme of phenylpropane synthesis pathway, PAL is often connected with plant resistant. Many studies have reported that PAL played positive function in plant resistant. Overexpression of a *PAL* gene from the https://www.sciencedirect.com/topics/agricultural-and-biological-sciences/tropical-pastures legume https://www.sciencedirect.com/topics/agricultural-and-biological-sciences/stylosanthes *humilis* in tobacco results in increased resistance to *Cercospora nicotianae* (Way et al., 2002). Overexpression of *Lotus japonicus PAL* (*LjPAL1*) delays the infection process and reduces the number of nodules after *Mesorhizobium loti* infection (Chen et al., 2017). Meanwhile, it has been demonstrated that PAL is involved in the biosynthesis of the signaling molecule salicylic acid, which is an organic acid necessary for the acquisition of resistance in plant systems (Lefevere et al., 2020).

Common wheat (*Triticum aestivum* L.) is the world’s major cereal crop, accounting for about 30% of the world’s staple food, it is also an important model tool for plant research (Zhu et al., 2015; Zhang et al., 2019). Given the resistant function of PAL, the role of PAL in wheat disease resistance was studied. The expression levels of *PAL* genes were up-regulated in wheat cultivars resistant to *Fusarium graminearum* (Golkari et al., 2009). Overexpression of *Aegilops variabilis PAL (AevPAL1)* significantly enhanced cereal cyst nematode resistance in bread wheat (Zhang et al., 2021). Although these studies further showed that the *PAL* genes played an important role in the process of stress defense, its comprehensive information and functions under biotic stress in wheat remains unclear. In this study, bioinformatics methods were used to identify and analyse *T. aestivum* PAL (TaPAL) family members and their characteristics. In addition, the expression patterns of *TaPALs* under disease stresses were also quantified. Our results provide important theoretical support for the functional study of wheat *PAL* genes, and also provide potential application value for high-yielding wheat varieties breeding against various biotic stresses.

## Materials and methods

### Identification of PALs protein sequences in the wheat

The wheat genome sequences IWGSC RefSeq v2.1 were downloaded from the International Wheat Genome Sequencing Association website (https://wheat-urgi.versailles.inra.fr/Seq-Repository/Assemblies) (Alaux et al., 2018). Firstly, the PAL domain sequence (Pfam accessions: PF00221) was downloaded from the Pfam database (http://pfam.xfam.org/), and were used as queries to search the PAL proteins in wheat genome by HMMER3.0 with cutoff values ≤ 1e-5 (Eddy, 2008; Finn et al., 2016). Secondly, the protein sequences of four *Arabidopsis* PALs (AtPALs), 18 maize PALs (ZmPALs), and nine rice PALs (OsPALs) were retrieved from the Arabidopsis Information Resource (TAIR10) database (http://www.arabidopsis.org/index.jsp), the Maize Genetics and Genomics Database (MaizeGDB) (https://www.maizegdb.org/), and the Rice Genome Annotation Project (RGAP) database (http://rice.plantbiology.msu.edu/), respectively (Huang et al., 2010; Zeng et al., 2018; Deng et al., 2019). Subsequently, the above mentioned 31 PAL protein sequences were used as queries to search wheat genome data through BLASTp, and the expected cutoff value was set as e ≤ 1e-5 to ensure the reliability of the protein sequence. After merging two rounds of search results and deleting redundant sequences, unique ones were further validated through SMART (http://smart.emblheidelberg.de/) and InterProScan (v71.0, http://www.ebi.ac.uk/InterProScan) to determine whether sequences contain PF00221 domain.

### Characterization of predicted TaPAL proteins

The protein characteristics of the TaPALs, including protein length, isoelectric point (pI), molecular weight (MW), instability index, atomic composition, grand average hydropathicity (GRAVY), and amino acid composition, were analyzed using ExPASy Server10 (SIB Bioinformatics Resource Portal, https://prosite.expasy.org/PS50011). The online tools TMHMM (http://www.cbs.dtu.dk/services/TMHMM/) and SignalP4.1 (http://www.cbs.dtu.dk/services/SignalP/) were used to predict the transmembrane domains and signal peptides in TaPALs (Nielsen, 2017). Subcellular localization prediction of TaPALs were performed using Plant-mPLoc online tool (http://www.csbio.sjtu.edu.cn/cgi-bin/PlantmPLoc.cgi) (Chou and Shen, 2010).

### Sequence alignment and phylogenetic tree construction

After collecting the protein sequences of four AtPALs, 18 ZmPALs, nine OsPALs, and TaPALs, DNAMAN (version 6.0) was used to align all amino acid sequences. The phylogenetic tree was constructed using the Maximum Likelihood (ML) method (1000 bootstrap trials) based on an LG model in MEGA7.0 software (Kumar et al., 2016). A midpoint rooted base tree was modified using the Interactive Tree of Life (IToL, version 5.5.1, http://itol.embl.de). The TaPALs were classified basing on the phylogenetic relationships.

### Gene structure, conserved motifs and cis-elements analysis of TaPALs

The *TaPAL* gene structure was exhibited using the online software GSDS V2.0 (http://gsds.cbi.pku.edu.cn/index.php) according to the wheat genome annotation information (Hu et al., 2014). Conserved motifs of TaPALs were predicted using the online program MEME V 5.1.1 (Multiple Expectation Maximization for Motif Elicitation, http://meme-suite.org/tools/meme) (Bailey et al., 2015). The parameters were as follows: each sequence could comprise any number of non-overlapping occurrences of each motif, the number of different motifs was 20, and motif length ranged from 6 to 50 amino acids (Fang et al., 2019). The conserved domains of these predicted motifs were analyzed using InterPro (http://www.ebi.ac.uk/interpro) and SMART (http://coot.embl-heidelberg.de/SMART). The PlantCARE (http://bioinformatics.psb.ugent.be/webtools/plantcare/html/) was used to predict cis-acting elements in the regions 1,500 bp upstream of the start codons of *TaPALs* (Lescot et al., 2002). TBtools software was used for combing and visualizing the above results (Chen et al., 2020).

### Chromosomal location, gene duplication, and Ka/Ks analysis

The gene annotation of *TaPALs* was extracted from wheat genome GFF3 file, and the chromosomal location was drawn with MapInspect software (Jiang et al., 2019). Gene duplications were divided into tandem and segmental replications. Tandem repeated events were identified using the following assessment criteria: (1) aligned sequence length > 80% regions of each sequence; (2) identity > 80%; (3) threshold ≤ 10^−10^; (4) only one duplication can be admitted when genes are linked closely; and (5) intergenic distance is less than 25 kb. If genes met the three conditions (1), (2) and (3) and were located on different chromosomes, they were judged as segmental duplications (He et al., 2020). The Ka (non-synonymous)/Ks (synonymous) ratio were calculated after identification of a duplicated gene, and the selection pressure and selection mode were analyzed. The CDS sequences of the duplicated gene pairs were compared using MEGA 7.0 software. After removal of gaps the alignment results were imported into DnaSP v5.10 software for Ks value and Ka/Ks ratio analyses (Librado and Rozas, 2009) . For estimation of the timing of duplication events the formula T = Ks/2λ × 10^-6^ Mya was used to calculate divergence time (T) in millions of years (Mya), where λ = 6.5 × 10^-9^ represented the rate of replacement of each locus per herb plant year (Quraishi et al., 2011; Peng et al., 2012). Duplicated gene pairs between species were identified and used to carry out an inter-species synthetic analysis using R package “circlize” (Krzywinski et al., 2009; Song et al., 2019).

### Inter-species evolution analysis of TaPALs

The genome sequence data and the annotation information of *Triticum urartu* (v1.43), *Triticum dicoccoides*, and *Aegilops Tauschii* (v4.0.43) were downloaded from Ensembl Plants database (http://plant.ensembl.org/index.html) (Bai et al., 2019). In order to better understand the origin of *TaPAL* genes, The PAL family genes in *T. urartu*, *T. dicoccoides*, and *A. Tauschii* were identified by the Blastp analysis. The cutoff values (e-value < 10^-10^, identity > 80%) were used to ensure the reliability of homologous (Li et al., 2022) . The original definition of an ortholog was two genes from two different species that were derived from a single gene of the last common ancestor of that species. Paralogs were defined as genes derived from a single gene that was repeated within the genome (Zhang et al., 2019). The synteny relationship were displayed with R package “circlize” and TBtools software (Zhu et al., 2015; Chen et al., 2020).

### Expression profiling of TaPAL genes in different tissues or under various stresses

Original data sets, including different development stages and tissues from variable treatments, were downloaded from the NCBI Short Read Archive (SRA) database and mapped to the wheat reference genome using Hisat2 (Li et al., 2022). The SRA numbers of those data were listed in **Supplementary file 1**. Cufflinks was used to calculate the expression levels of *TaPALs* (normalized by Transcripts Per Kilobase of exon model per Million mapped reads, TPM). The R package “pheatmap” was used to produce the heatmap.

### Growth and stress treatment of wheat seedlings

The hexaploid common wheat cultivars Jingshuang 16 and Yangmai 158, were moderately sensitive to *Puccinia striiformis* f. sp. *tritici* and *F. graminearum*, respectively. Full-size seeds were surface disinfected with 1% hydrogen peroxide, and thoroughly rinsed with distilled water, then germinated in 25°C saturated water on three-layer filter paper (Zhang et al., 2019). The growth conditions were 25/20°C temperature and 16 h/8 h (day/night) photoperiod. The wild type strain of *F. graminearum* (PH-1) was cultivated on PDA plate and cultured at 25°C for 3 days. And then, the actively growing mycelial agar plugs (5 mm diameter) were transferred to a 150 mL conical flask containing 50 mL of liquid mung bean medium, and was cultured at 25°C with 150rpm for 3 days to produce conidia (Cao et al., 2016). After the coleoptile reached to 0.5 cm, the seedlings of Yangmai 158 were immersed in spore suspension (5 × 10^5^ spores/mL) for 1 minute (Yang et al., 2010). Then the seedlings were wrapped in a wet filter paper and grown at 25°C with 70% relative humidity. The uninoculated wheat seedlings were used as control. The wheat stems were sampled at 6, 12, 24, and 48 h after inoculation.

The *P. striiformis* was remained on the susceptible cultivar Jingshuang 16. At two-leaf stage, the seedings were sprayed with fresh sporangium powder of CYR23 (4 mg/mL) and kept in dark place for 24 hours with moisture. The seedlings were cultured in 10-18°C light for 16 h/8 h (day/night) photoperiod. Wheat leaves were sampled at 6, 12, 24, and 48 h after inoculation. The uninoculated wheat was used as a control. The samples were immediately frozen liquid nitrogen and stored at -80°C. Each treatment included three technical replications and three biotic replicates in each replication.

### Quantitative real-time PCR and data analysis

After analyzing the transcriptome data, five *TaPAL* genes (*TaPAL10, TaPAL14, TaPAL32, TaPAL42, TaPAL49*) with high expression levels were screened out. The expression levels of these five genes under *F. graminearum* and *P. striiformis* infection were analyzed by qRT-PCR. Total RNAs were reverse transcribed into cDNAs with a 5 × All-In-One RT Master Mix (Perfect Real Time) kit (ABM, Canada). Gene-specific primers were designed using Primer Premier 5.0 and listed in **Supplementary file 1**. For qRT-PCR assays, the cDNA was diluted into 100 ng/μL with ddH_2_O. The 20 μL reaction volume contained 10 μL of 2 × SYBR Green Mix, 1 μL of each primer (10 μM), 2 μL of template, and 6 μL of ddH_2_O. All quantitative real-time PCR (qRT-PCR) amplifications were performed on the iCycler iQ instrument (Biorad, Hercules, CA, USA). The following cycling parameters were used: initial denaturation at 95°C for 2min; 50 amplification cycles consisting of denaturation at 95°C for 15s, annealing and extension at 57°C for 45 s. Single-fragment amplification was verified by dissociation curve analysis. Three biotic replicates were performed for each sample, and each replicate contained three technical replicates. ADP-ribosylation factor Ta2291 was used as internal reference gene (Paolacci et al., 2009). Relative expression levels were calculated using the 2^-ΔΔCt^ method (Livak and Schmittgen, 2001) . The heatmap were plotted using Origin software (Moberly et al., 2018).

### Subcellular localization of TaPAL proteins

To verify the prediction results of TaPAL proteins localization, the CDS region of *TaPAL32* and *TaPAL42* were amplified using cDNA as template. The vector pART27:GFP was digested with *Xho*I (Biomarker, China) and then purified by Cycle-pure Kit (Omega, UK). The full-length gene fragments were inserted into the purified pART27:GFP vector using ClonExpress II one-step cloning kit (Vazyme, China). Then the recombinant vector pART27:TaPAL32*-*GFP and pART27:TaPAL42-GFP were transformed into *Agrobacterium tumefaciens* GV3101. The leaves from 6 to 8 week-old *Nicotiana benthamiana* were injected with *A. tumefaciens*. After 48 h, the GFP signals were detected and were observed under fluorescence microscope (Nikon DS-Ri2, Japan).

### BSMV-mediated gene silencing

The spring wheat cultivar Fielder was used in this experiment. Approximately 120bp of gene fragments of *TaPAL32* and *TaPAL42* were amplified from wheat cDNA using the primers listed in **Supplementary file 1**. Then the fragments were inserted into the BSMV:γ vector. The virus was inoculated on the second leaf of the two-leaf wheat seedlings by mechanical friction method and then placed in an artificial incubator for 25°C cultured. BSMV : TaPDS (TaPDS, wheat octahydrolysin desaturase) was used as a positive control, and 1× Fes buffer (0.1M glycine, 0.06M K2HPO4, 1% w/v tetrasodium pyrophosphate, 1% w/v bentonite, and 1% w/v celite, pH 8.5) treated plants were used as negative control (Mock) (Zhu et al., 2018). At 10 dpi, the leaves with mosaic symptoms were inoculated with fresh uredospores of *P. striiformis* (CYR23). Inoculated leaves were collected at 0, 24, and 48 h after inoculation to determine the efficiency of silencing. The experiment was repeated at least three times. Relative expression levels were determined using the 2^–ΔΔCt^ method (Livak and Schmittgen, 2001).

## Results

### Identification and characterization of PAL proteins in wheat

Firstly, 571 wheat candidate PAL proteins were obtained by Blastp analysis. Then, 65 candidate PAL proteins were screened in the wheat genome through SMART and InterProScan. Finally, the results were combined to remove redundant sequences, and 54 PAL members were identified in wheat genome. These TaPAL proteins ranged from 265-714 amino acids in length, with predicted molecular weights ranging from 29.80 kDa to 77.34 kDa (**Supplementary file 2**). The isoelectric points of 51 TaPAL proteins were less than 7. Only the pI values of TaPAL14, TaPAL26, and TaPAL43 had were greater than 7. The grand average of the hydrophilicity (GRAVY) of all TaPALs were less than zero, suggesting that they were almost all hydrophilic proteins. The proteins stability prediction results showed that all TaPALs were unstable proteins. Subcellular localization predicted results showed that all TaPALs were located in the cytoplasm.

### Phylogenetic analyses of TaPALs

**Figure 1:**
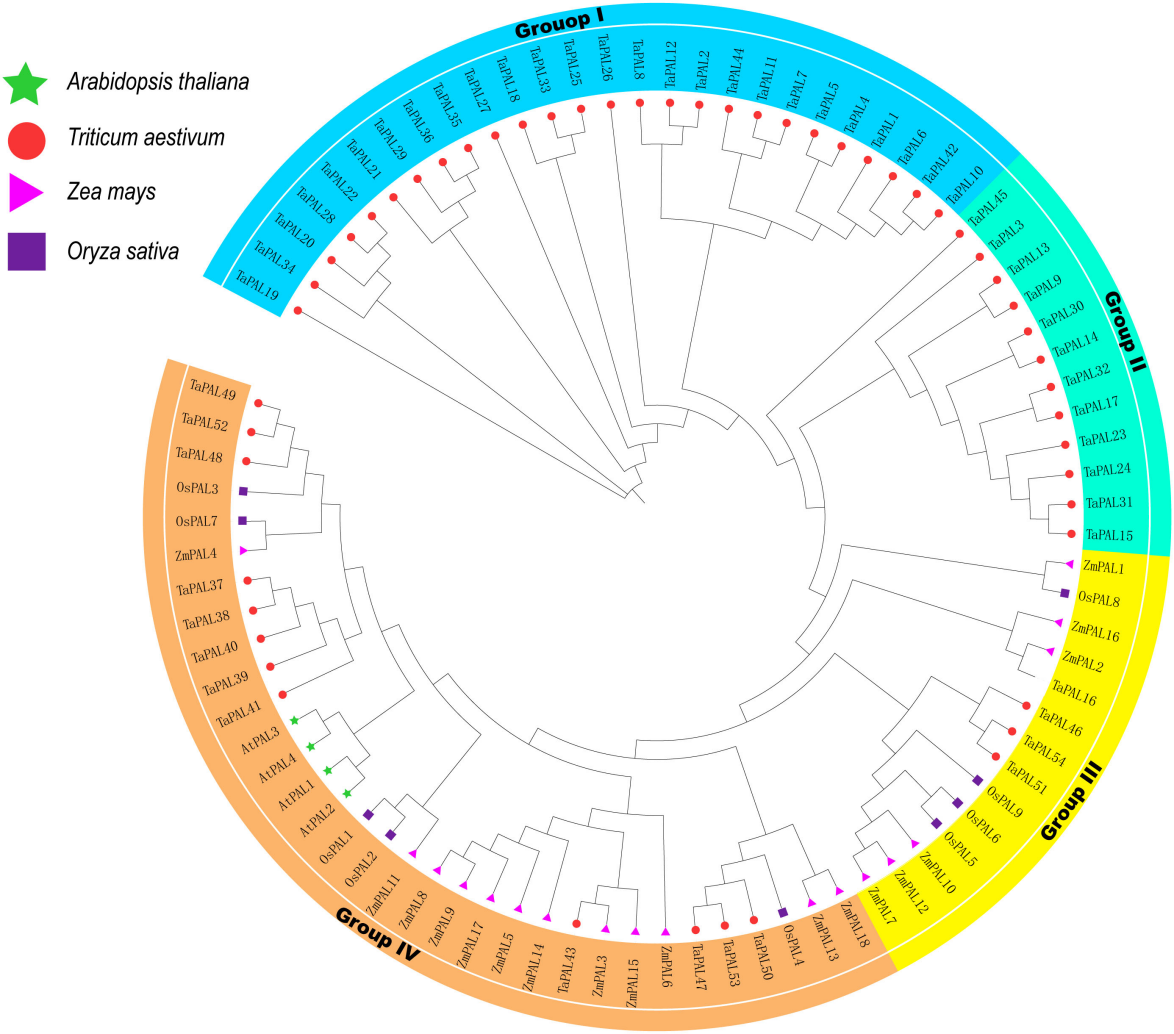
Phylogenetic relationship of TaPALs, OsPALs, AtPALs, and ZmPALs. Protein sequences were aligned using ClustalW2 sequence alignment program and the phylogenetic tree was constructed by software MEGA7 used to create Maximum Likelihood (ML) under the LG model. The tree was constructed with 1,000 bootstrap replications. Different groups were marked by different colors, and the PAL from wheat, rice, maize, and *Arabidopsis* were distinguished with different color and shape.

To understand the evolutionary relationships of these 54 TaPAL proteins, a maximum likelihood phylogenetic tree was built based on all PAL proteins in wheat, maize, rice, Arabidopsis (**Supplementary file 3**). the phylogenetic tree showed that all PAL proteins were divied into four Groups (Group I, II, III, and IV) (**Figure 1**), which was consistent with the previous classification (Lepelley et al., 2012; Yan et al., 2019). Overall, Group I contained the largest number of TaPALs (26) and Group III had the lowest number of TaPALs (four).

### Analysis of gene structure, protein motifs and sequence conservation for TaPALs

**Figure 2:**
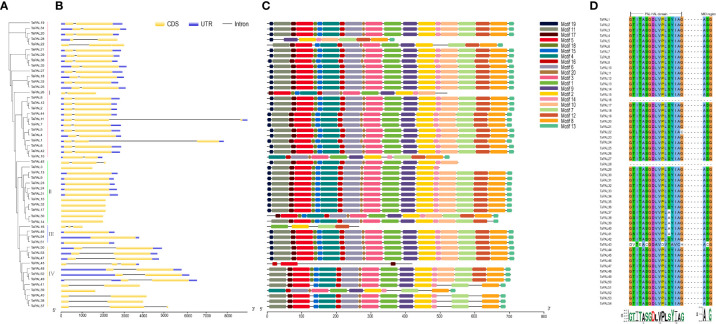
Phylogenetic analysis, gene structure, conserved motifs and sequence alignment of PAL domains of TaPALs. **(A)** The phylogenetic tree of all *PAL* genes in common wheat. The tree was created with bootstrap of 1000 by maximum likelihood (ML) method in MEGA7. **(B)** The exon-intron structure of *PAL* genes in common wheat. **(C)** The motif compositions of TaPAL were identified by MEME. Model exhibition of motif compositions in PAL amino acid sequences using MAST. **(D)** Multiple sequence alignment of PAL domains in identified wheat PAL proteins.

In order to understand gene structure of TaPALs, we analyzed wheat GFF3 format annotations. The results showed that these genes contain no more than two introns, and 12 TaPAL genes were intronless. More interestingly, most of these genes were clustered into Group II and III (**Figure 2**). In Group I, TaPAL1 and TaPAL11 contained two introns, while the remaining members had only one intron. Compared with genes in the other three groups, the exon-intron structures of genes in Group IV were more complicated. TaPAL39 had no intron, three TaPAL genes (TaPAL37, TaPAL43, and TaPAL49) had two introns, and the remaining members had only one intron.

In order to study the conserved regions of TaPAL proteins, the motifs of 54 TaPAL proteins were analyzed by MEME online software. Twenty conservative motifs were identified (**Figure 2** and **Supplementary file 4**). Eight of these motifs (motif1-2, 4-6, and 10-12) were found to be associated with characteristic functional domains of the typical plant PAL protein. Motif 1 was mostly present in most of TaPAL proteins. Analysis of conserved motifs showed that TaPAL protein domains were distributed in almost all of the 54 members.

Due to the high sequence homology of PAL in plants, the protein sequences of 54 TaPAL were aligned. As shown in **Figure 2**, most TaPAL proteins had conserved enzyme active sites in the PAL-HAL and MIO domains, except for the nine core domains of TaPAL16, TaPAL22, TaPAL28, TaPAL37 to 41, and TaPAL43. It was worth noting that TaPAL16 and TaPAL28 lacked the core domains of PAL-HAL and MIO. In the MIO region, two conserved residues, Ala and Gly, were found to be conserved in all TaPAL proteins containing this domain. Although the PAL-HAL domain was conserved among 54 TaPALs, Gly residues had been replaced by Cys and His in TaPAL22 and TaPAL43, respectively. These analyses indicated that most TaPAL proteins may have enzymatic activity, but their activities were different.

### Chromosome distribution, gene duplication events, homology analysis, and Ka/Ks analysis

**Figure 3:**
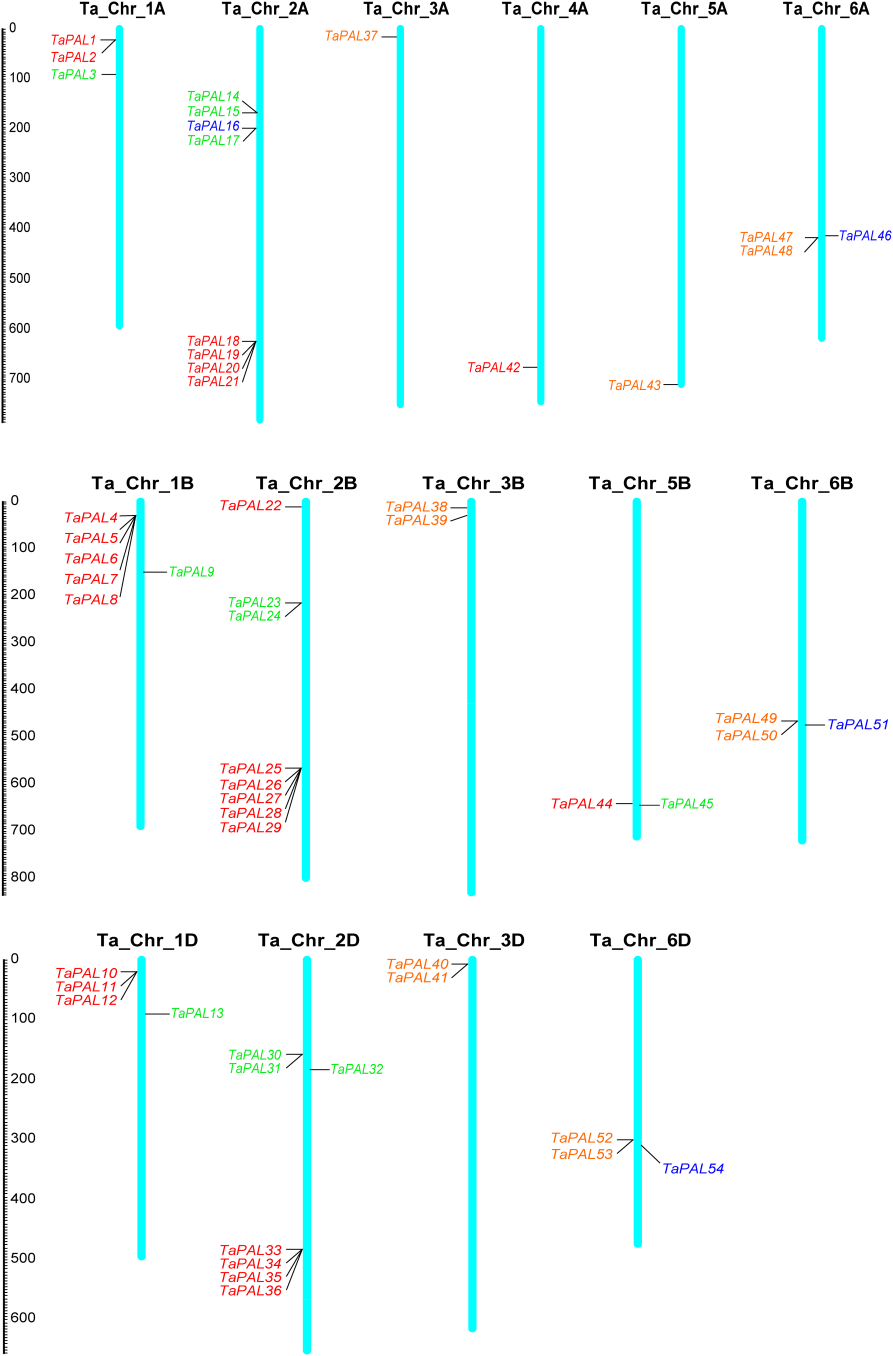
Chromosomal localization of the 54 TaPALs in wheat genome. Different classes of TaPALs are represented in different colors. Red represents TaPAL-I, green represents TaPAL-II, blue represents TaPAL-III, and orange represents TaPAL-IV. In addition, Ta represents wheat (*Triticum aestivum*), Chr represents Chromosome.

**Figure 4:**
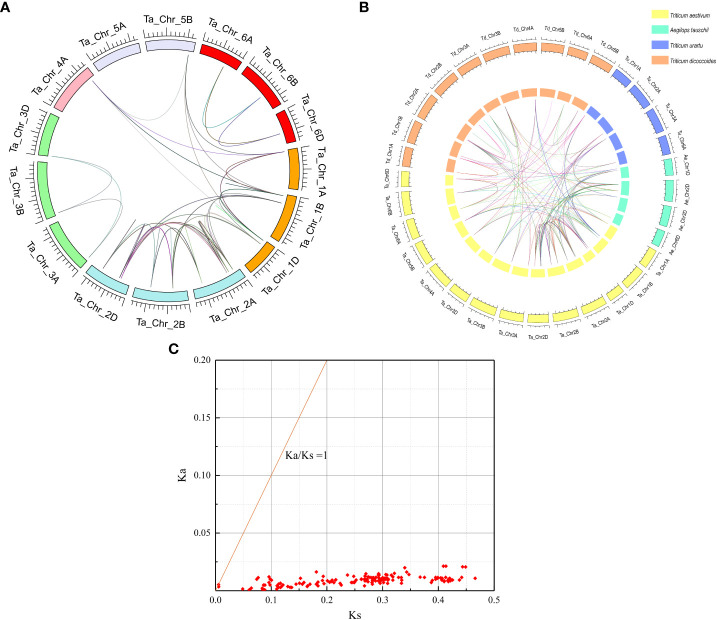
Whole genome duplication (WGD)-derived and homology relationship analysis of wheat PAL genes. **(A)** Analysis of genome-wide *PAL* genes replication events in common wheat (Ta, AABBDD). Different colors represent different chromosomes; WGD/segmentally duplicated *TaPAL* gene pairs are linked by different colored lines. **(B)** Homology relationship analysis of the *TaPAL* genes of *Triticum aestivum* and its subgenome donors *Triticum urartu*, *Triticum dicoccoides*, and *Aegilops tauschii*. The reference genomes of *T. urartu* (v. 1.43), *T. dicoccoides* (v. 1.0.43), and *A*. *tauschii* (v. 4.0.43) were downloaded from the Ensembl Plants database (http://plants.ensembl.org/index.html). The orange box represents *T. dicoccoides*, the blue box represents *T. urartu*, the cyan box represents *A. tauschii* and the yellow box represents *T. aestivum*. **(C)** Ka/Ks values for duplicated *TaPAL* genes pairs.

Chromosome mapping showed that 54 *TaPAL* genes were unevenly distributed on 16 chromosomes of common wheat (**Figure 3** and **Supplementary file 5**). *TaPALs* were mainly distributed on chromosomes 2A, 2B and 2D, which contained eight, eight and seven genes, respectively. It was the least distributed on chromosomes 3A, 4A and 5A, and there was only one gene on each chromosome. In order to adapt to different environmental conditions, tandem replication and fragment replication were necessary for the evolution of gene families, and duplicate events of *TaPALs* need to be detected. As shown in **Figure 4** and **Supplementary file 6**, 29 pairs of tandem duplication genes and 115 pairs of segment duplication genes were identified in 54 *TaPALs*, and most interestingly, the tandem repeat genes were only found in Group I and II.

In order to further infer homology of wheat PAL family, a total of 91 PALs were identified from T. aestivum (45 TaPALs), T. urartu (9 TuPALs), T. dicoccoides (26 TdPALs) and Ae. tauschii (11 AePALs) by a computer-based method (**Figure 4**). After statistics, there were 393 pairs of orthologous gene pairs, 78 orthologous gene pairs were identified from T. aestivum and Ae. tauschii, 33 orthologous gene pairs were identified from T. aestivum and T. dicoccoides, and 66 orthologous gene pairs were identified from T. aestivum and T. urartu. In addition, T. aestivum, T. urartu, T. dicoccoides and Ae. tauschii had 146, 7, 25 and 6 pairs of paralogous genes respectively.

We further analyzed the Ka (nonsynonymous)*/*Ks (synonymous) ratio of replicated PAL genes pairs in wheat to understand the evolutionary constraints on TaPALs (**Figure 4** and **Supplementary file 7**). In general, Ka/Ks rates can be used to evaluate the selectivity of coding sequences; the Ka/Ks ratio *>*1 indicates accelerated evolution with positive selection, a ratio =  1 indicates neutral selection, and a ratio < 1 indicates negative or purifying selection (Zhang et al., 2006; Peterson and Masel, 2009). The Ka/Ks ratios of 144 replicated gene pairs were calculated. The Ka/Ks ratios for all these TaPALs duplicated gene pairs were less than 1, which indicated that all repeated genes of the TaPAL family have undergone strong purifying selections which may help maintain their functional stability.

### Analysis of cis-regulatory elements in the promoter region of TaPAL genes

**Figure 5:**
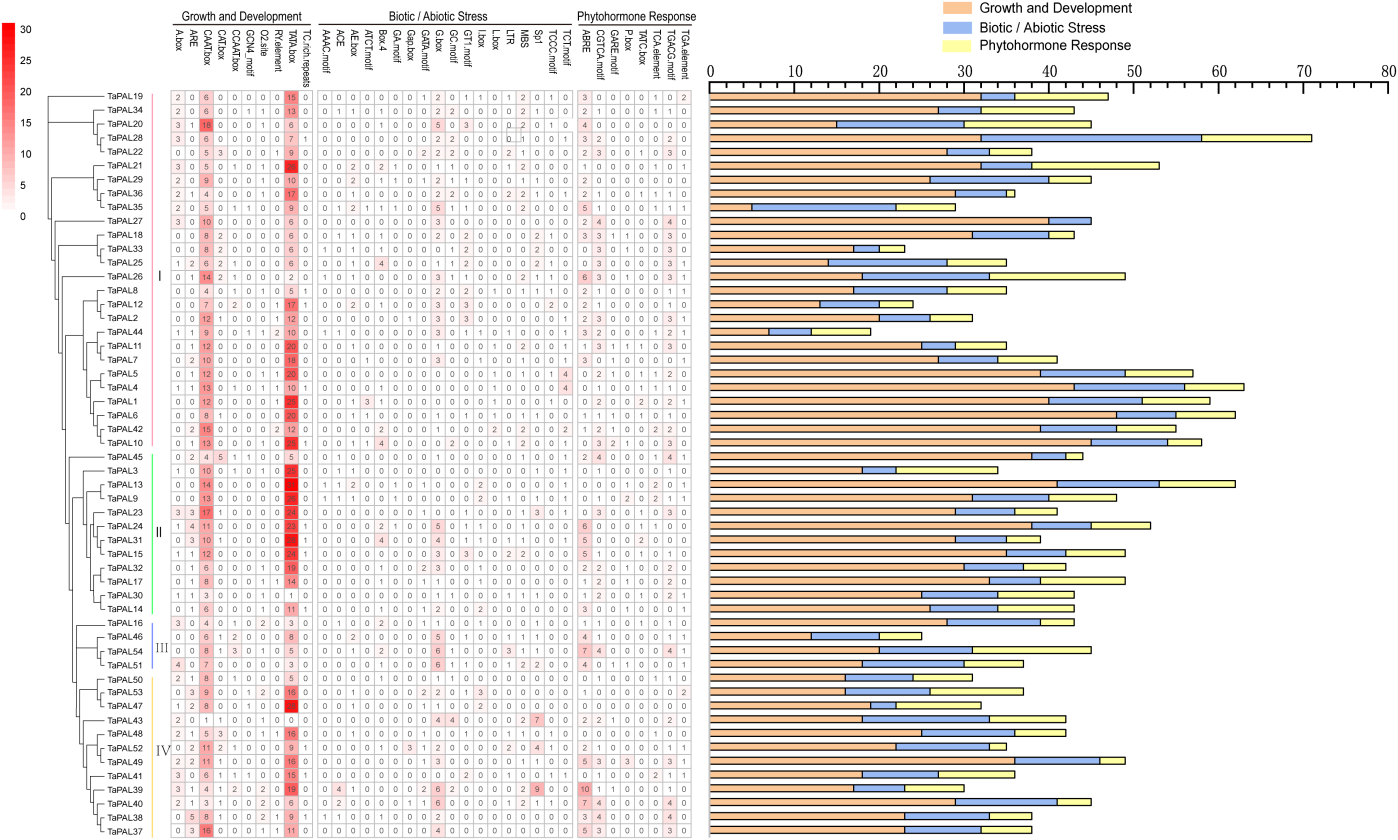
Analysis of cis-acting element numbers in TaPAL genes promoters. The different colors and numbers of the grid indicated the numbers of different promoter elements in these TaPAL genes. The different colored histogram represented the sum of the cis-acting elements in each category.

The cis-regulatory element was specific motif that can be combined with appropriate transcription factors to further regulate the transcription of genes in plants (Wittkopp and Kalay, 2011). To identify putative cis-elements in the TaPAL gene promoter region, we analyzed the 1500 bp upstream promoter region of TaPAL genes. We found that a total of 2292 cis-acting elements were predicted, of which 1422 regulatory-elements were related to growth and development, 485 regulatory elements were related to biotic and abiotic stress, and 385 active-elements were related to phytohormone (**Figure 5** and **Supplementary file 8**). As shown in **Figure 5**, growth-related cis-regulatory elements include zein metabolism regulation (O2-site), seed-specific regulation (RY-element), etc. Furthermore, cis-regulatory elements related to stress included light responsive elements (ACE, GT1-motif, AE-box, ATCT-motif, and Box 4), enhancer-like element involved in anoxic specific inducibility (GC-motif), low-temperature responsiveness (LTR). The remaining cis-elements were related to phytohormone response, included cis-acting element involved in the abscisic acid responsiveness (ABRE); gibberellin-responsive element (GARE-motif and P-box). We found that CAAT box and TATA box were the most common cis-acting elements in TaPALs. Meanwhile, many cis-acting elements were related to plant abscisic acid (ABRE), which was distributed in the promoter region of most of TaPALs. Cis-element analysis showed that TaPAL family members may be play an important role in transcriptional regulation and abscisic acid metabolism pathway.

### Transcriptional analysis of TaPAL genes

**Figure 6:**
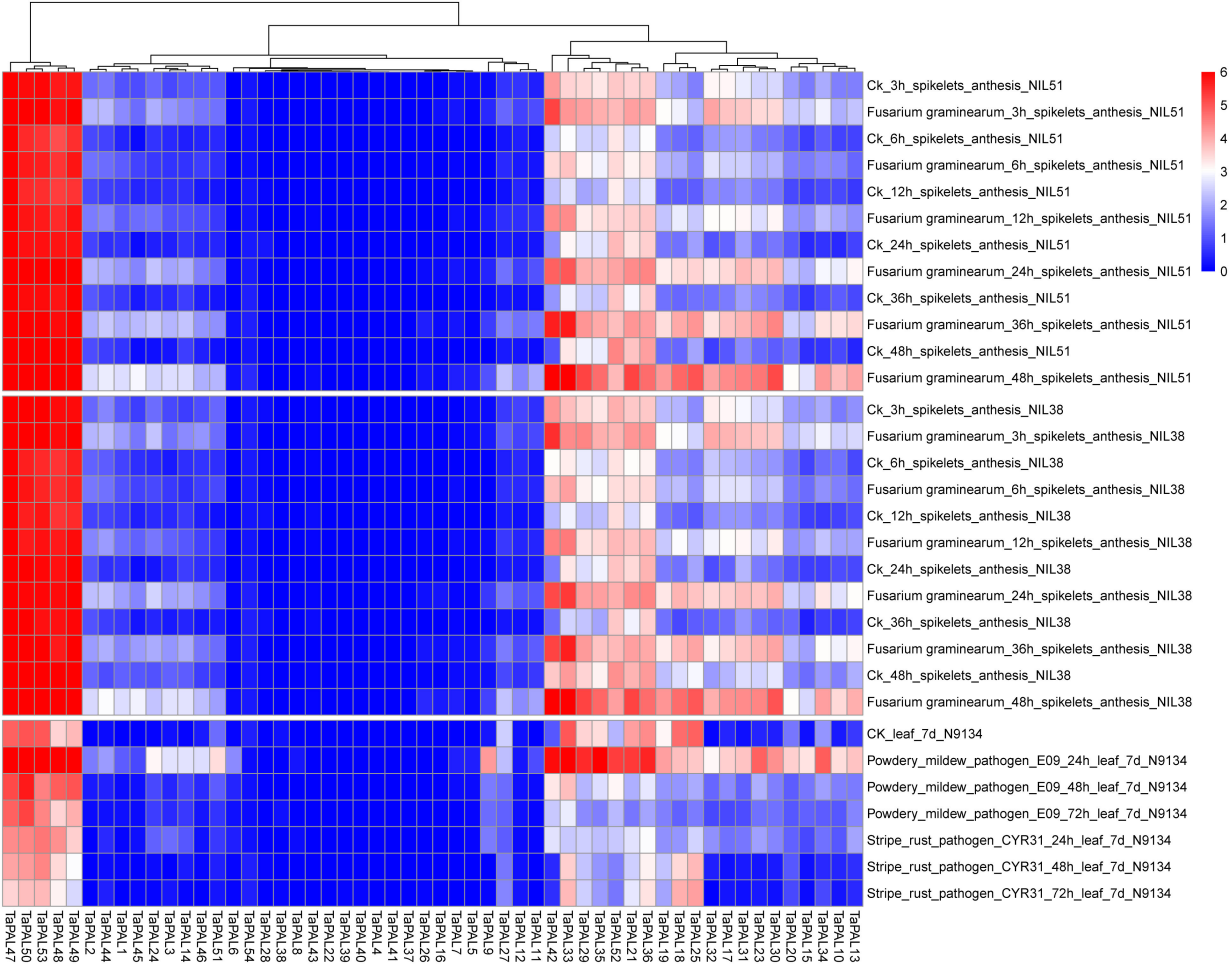
Heat map of expression profiles for TaPAL genes across different stresses under different biotic stress. Blocks with colors indicate decreased (blue) or increased (red) expression levels. The gradually change of the color indicates different level of gene log2-transformed expression (fold change > 3 is significantly expressed).

RNA-seq is a powerful tool to explore gene transcription patterns using high-throughput sequencing methods (Wang et al., 2010). Transcriptome data of wheat inoculated with F. graminearum, P. striiformis, and powdery mildew were used for analysis. In the expression profile (**Figure 6**), 15 TaPALs were not expressed and five TaPALs (TaPAL47, TaPAL48, TaPAL49, TaPAL50, TaPAL53) were generally highly expressed in various periods. The expression levels of 31 TaPALs were up-regulated and the expression level of TaPAL52 was down-regulated after inoculation with F. graminearum. The expression levels of 11 TaPALs were up-regulated and 14 TaPALs were down-regulated after inoculation with P. striiformis. Moreover, the expression levels of 32 TaPALs were higher in powdery mildew treated leaves than that in the control group, while the expression levels of TaPAL18, TaPAL25 and TaPAL27 were lower than that in the control group. These results indicated that TaPALs had different expression patterns under different disease treatments. Under the three stress conditions, the expression levels of ten TaPALs (TaPAL3, TaPAL10, TaPAL13, TaPAL14, TaPAL17, TaPAL30, TaPAL31, TaPAL32, TaPAL42, TaPAL48) were highly induced. It was inferred that these genes may be involved in the regulation of plant disease resistance.

### Quantitative real-time PCR analysis of wheat PAL genes in responses to different treatments

**Figure 7:**
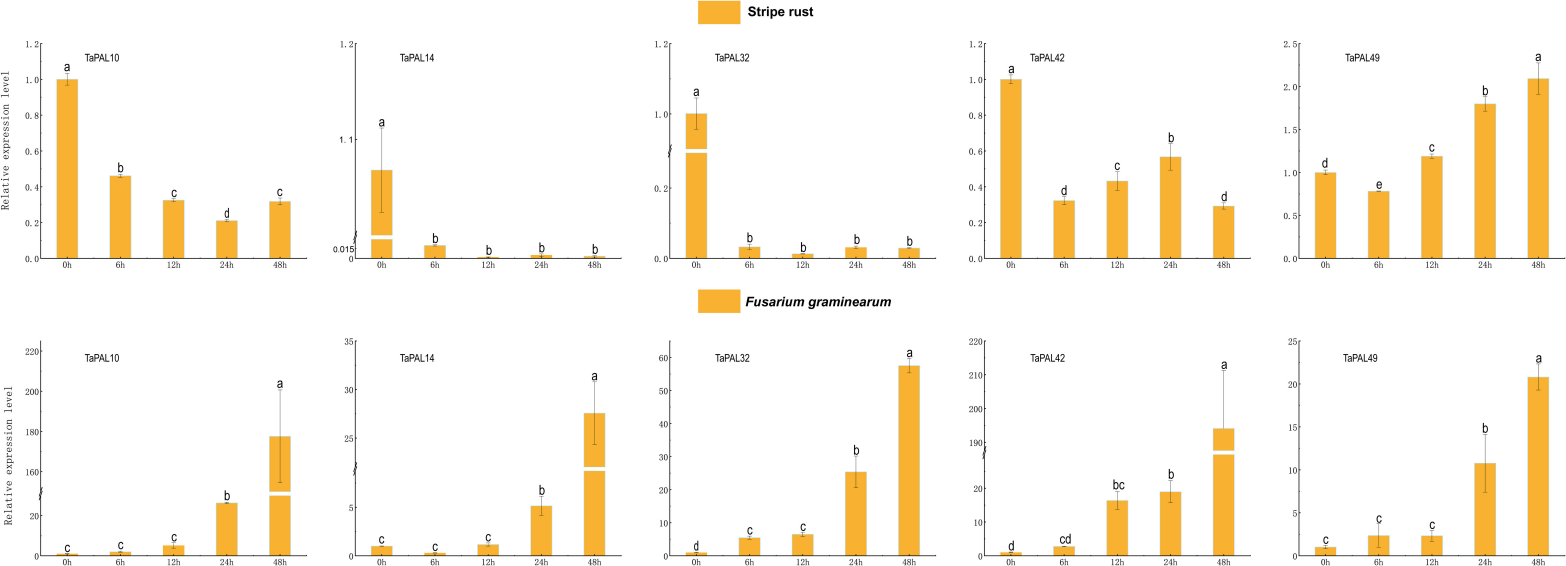
Reverse transcription quantitative PCR analysis of five TaPAL genes under, *F. graminearum*, and *P. striiformis* treatment. Each bar represents the average of three replicates, and error bar represents standard deviation (SD). Different letters in a column indicate significant differences between the treatments at p < 0.05 level.

In order to further explore the potential role of five genes (TaPAL10, TaPAL14, TaPAL32, TaPAL42, TaPAL49) under biotic stresses (F. graminearum and P. striiformis), we analyzed their expression levels by qRT-PCR. As shown in **Figure 7**, the expression levels of five genes were continuously up-regulated from 12* h* to 48* h* under F. graminearum treatment, among which only the expression levels of TaPAL32 and TaPAL42 were significantly up-regulated from 0* h* to 6* h*. In wheat leaves treated with P. striiformis, the expression levels of TaPAL10, TaPAL14, TaPAL32, and TaPAL42 showed a general downward trend. However, the expression level of TaPAL49 at 48* h* was much higher than that at 24* h*, and the expression level was gradually up-regulated during infection.

### Subcellular localization of TaPALs

**Figure 8:**
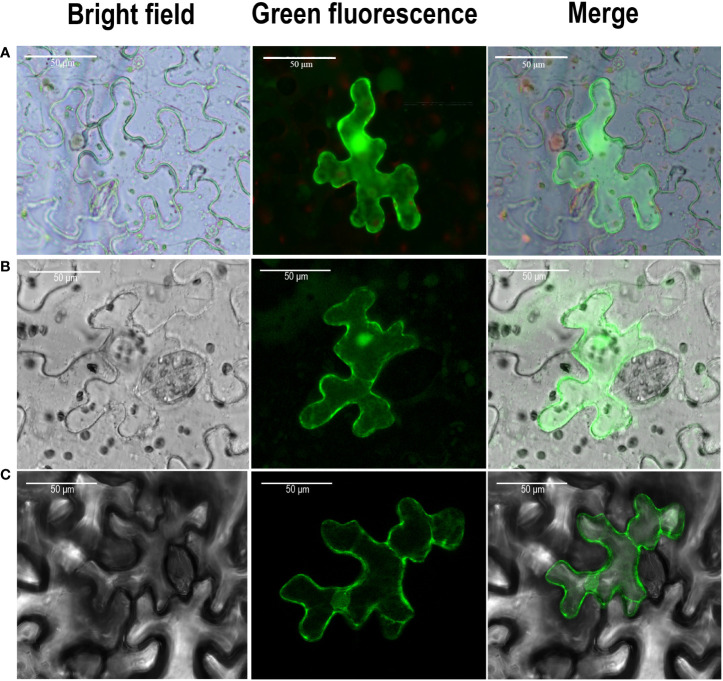
Subcellular localization of TaPAL genes. **(A)** Subcellular localization of free GFP. **(B)** Subcellular localization of TaPAL32. **(C)** Subcellular localization of TaPAL42. Subcellular localization was then observed by confocal laser scanning microscopy 48 – 72 h after infiltration. Scale bar = 50 μm.

The predicted results from plant-MPLOC online tool showed that TaPAL proteins were located in the cytoplasm. To further confirm the predicted results, TaPAL32-GFP and TaPAL42-GFP fusion protein were transiently expressed in *N. benthamiana* leaves. As shown in **Figure 8**, both of TaPAL32 and TaPAL42 were expressed in the whole cell.

### Silencing of TaPALs enhanced the sensitivity of wheat against Puccinia striiformis infection

**Figure 9:**
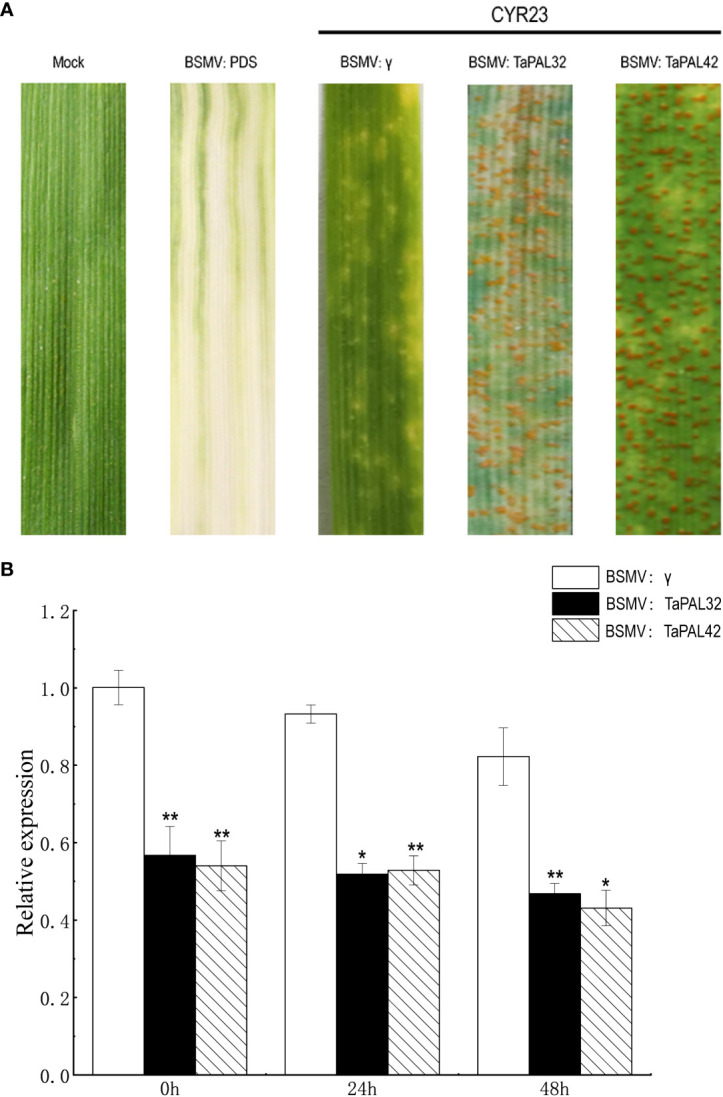
Silencing of TaPAL32 and TaPAL42 reduce the resistance of wheat to *P. striiformis*. **(A)** BSMV: TaPDS showed photobleaching at 10 dpi; Mock: wheat leaves treated with 1× Fes buffer. Disease symptoms on the leaves challenged with *Puccinia striiformis f.* sp. *tritici* race CYR23. Disease symptoms were photographed at 14 days post inoculation. **(B)** Silencing efficiency assessment of *TaPAL32* and *TaPAL42* in the 0, 24, and 48 h. *TaPAL32* and *TaPAL42* silenced plants treated with *P. striiformis* (*p < 0.05, **p < 0.01).

We further detected the function of *TaPAL32* and *TaPAL42* in the wheat cultivar Fielder by barley stripe mosaic virus (BSMV)-mediated virus-induced gene silencing (VIGS) assay. The silenced vectors of two genes (BSMV : *TaPAL32*, BSMV : *TaPAL42*) were constructed. About 10 days after BSMV treatment, BSMV : TaPDS leaves showed symptoms of photobleaching, indicating that the virus-induced gene silencing system was working. Then, fresh urediospores of *P*. *striiformis* isolates CYR23 was inoculated on the fourth leaves of silenced plants. The expression levels of *TaPAL32* and *TaPAL42* at 0, 24, and 48 h after inoculation were analyzed by qRT-PCR. Compared with the control (BSMV:γ), the gene expression levels of *TAPAL32* and *TaPAL42* were significantly down-regulated, and the disease severity was photographed at 14 days after inoculation. The results showed that there were many urediospores on the leaves of silent plants compared with the control group (**Figure 9**). It indicated that silencing of these two genes weakened the wheat defense responses against *P*. *striiformis.*

## Discussion

Phenylalanine ammonia lyase (PAL) plays an important role in the metabolic pathway of plant disease resistance substances such as lignin and phytoprotectin. When plants encounter adversity or disease, the expression of *PAL* gene can adapt to adversity or resist pathogen invasion by participating in synthesizing important secondary metabolites through phenylpropanoid metabolism (de Jong et al., 2015). At present, *PAL* genes have been isolated from many species. However, the role of PAL family in wheat has not been systematically studied. In this study, we identified 54 *TaPAL* genes in wheat genome. The result was significantly higher than the 37 *TaPALs* already reported in wheat. Because Rasool’s screening conditions were more stringent, two screening conditions (sequence identity greater than 75% and value ≤ 1e-10) were set to identify the wheat PAL family members (Rasool et al., 2021) . TaPAL family can be divided into four groups based on phylogenetic analysis. In the process of plant evolution, multiplication and variation of an ancestor can produce multiple family members, which may be clustered on one chromosome or distributed on different chromosomes (Whetten and Sederoff, 1992). The *TaPAL* genes were located across 15 chromosomes, with three of them having the most genes: seven on 2A and 2D, and eight on 2B. The PAL family members are unevenly distributed on the chromosomes of wheat, which may be related to genome replication and chromosome duplication.

Phenylalanine ammonia-lyase is widely present in plants. The TaPAL proteins have different physical and chemical properties, acid base, molecular weight and coding length, indicating that *TaPALs* have a high genetic diversity. With the gradual enhancement of species evolution and environmental adaptability, the genetic variation of species has been enriched. So genetic variation is the root cause of genetic diversity (Ohta, 1980). In TaPAL family, there are differences in cis-elements among family members, which may be caused by genetic variation during evolution. The undetectable expression of *TaPAL43* in different treatments of wheat may be due to the few CAAT boxes and TATA boxes. This result suggests that the different regulation of *TaPALs* expression may be due to the different cis-elements contained in each family member.

The products of phenylpropane metabolic pathway play a crucial role in plant growth and development and response to stress (Harakava, 2005). *PAL* genes are involved in plant response to pathogens (Alvarez et al., 2013). In *Arabidopsis thaliana*, the 4 knockout mutant of pal1/pal2/pal3/pal4 shows the phenotype of dysplasia and sterility, and is susceptible to *Pseudomonas syringae* (Huang et al., 2010). Transcriptome data showed that many *TaPAL* genes were up-regulated in *F. graminearum* and *P*. *striiformis* infection. Five *TaPALs* (*TaPAL10*, *TaPAL14*, *TaPAL32*, *TaPAL42*, *TaPAL49*) were screened by qRT-PCR analysis. The expression levels were significantly up-regulated under the stress of *F. graminearum*, which was consistent with the expression analyzed by transcriptome. However, except for *TaPAL49*, all *TaPALs* were down regulated under *P*. *striiformis* infection. This may be related to our selection of different wheat varieties for real-time PCR experiment. Transcriptome data showed that the wheat line N9134 was used as experimental material for *P*. *striiformis* infection. But, the susceptible wheat cultivar Jingshuang 16 was used for qRT-PCR. The expression of disease-resistant genes may also down-regulated during the process of disease infection. It had been confirmed that *GhERF5-4D* in cotton was up-regulated in resistant varieties and down-regulated in susceptible cultivars, the silenced lines were more susceptible after gene silencing (Zhao et al., 2022). Subsequently, we silenced two genes (*TaPAL32* and *TaPAL42*) using VIGS to further verify the role of *TaPALs* in wheat stripe rust resistance. The silencing efficiency results showed that the expression levels of *TaPAL32* and *TaPAL42* decreased by about 45% compared with the control group at 0, 24 and 48 h after inoculation. At 14 days after inoculation, the symptoms of silent plants were more severe than those of control plants, indicating that silencing of these two genes resulted in decreased disease resistance of wheat. These results strongly proved that members of TaPAL family were involved in the infection of wheat stripe rust, but the mechanism of *TaPAL32* and *TaPAL42* may be complex and needs further study.

## Conclusion

In this study, 54 *PAL* genes were identified from the whole genome of wheat, and their phylogenesis, chromosome distribution, conserved cis-acting elements and gene expression profile under biotic stress were characterized. More importantly, gene silencing results showed that PAL family members play an important role in wheat resistance to *P. striiformis* infection. In conclusion, our findings provide new clues for improving disease resistance in wheat. This study provides a reference for subsequent studies on the function of TaPAL family.

## Data availability statement

The original contributions presented in the study are included in the article/**Supplementary Material**. Further inquiries can be directed to the corresponding authors.

## Author contributions

YaL and DM designed this article; CZ and YaL directed the data analysis and manuscript writing. YiL, MW and HL supervised the experiment. SG confirmed the manuscript. All authors contributed to the article and agreed to submit version of the manuscript.

## Funding

This research was supported by “Open Program of Engineering Research Center of Ecology and Agricultural Use of Wetland Ministry of Education” (KFT202103)”, and “Open Project Program of Key Laboratory of Integrated Pest Management on Crop in Central China, Ministry of Agriculture/Hubei Province Key Laboratory for Control of Crop Diseases, Pest and Weeds (2021ZTSJJ8).

## Conflict of interest

The authors declare that the research was conducted in the absence of any commercial or financial relationships that could be construed as a potential conflict of interest.

## Publisher’s note

All claims expressed in this article are solely those of the authors and do not necessarily represent those of their affiliated organizations, or those of the publisher, the editors and the reviewers. Any product that may be evaluated in this article, or claim that may be made by its manufacturer, is not guaranteed or endorsed by the publisher.
